# Dopamine D_2/3_R availability after discontinuation of antipsychotic treatment: a [^11^C]raclopride PET study in remitted first-episode psychosis patients

**DOI:** 10.1017/S003329172510161X

**Published:** 2025-09-08

**Authors:** Franciska de Beer, Erik de Vries, Ben Wijnen, Marieke J.H. Begemann, Nico van Beveren, Nynke Boonstra, Shiral S. Gangadin, Lieuwe de Haan, Iris M.H. Hamers, Wim Veling, Sanne Koops, Iris E.C. Sommer

**Affiliations:** 1 University of Groningen, University Medical Centre Groningen, Center for Clinical Neuroscience and Cognition, Groningen, The Netherlands; 2 University of Groningen, University Medical Centre Groningen, Department of Nuclear Medicine and Molecular Imaging, Groningen, The Netherlands; 3 Centre of Economic Evaluations & Machine Learning, Trimbos Institute, Netherlands Institute of Mental Health and Addiction, Utrecht, The Netherlands; 4 Parnassia Group for Mental Health Care, The Hague, The Netherlands; 5 Department of Neuroscience, Erasmus Medical Centre, Rotterdam, The Netherlands; 6 Department of Healthcare, NHL Stenden University of Applied Sciences, Leeuwarden, The Netherlands; 7 KieN Early Intervention Service, Leeuwarden, The Netherlands; 8 Department of Psychiatry, UMC Utrecht Brain Center, Utrecht, The Netherlands; 9 Department of Psychiatry, Amsterdam University Medical Centre – location AMC, University of Amsterdam, Amsterdam, The Netherlands; 10 Department of research, Arkin Mental Health Care, Amsterdam, The Netherlands; 11 University of Groningen, University Medical Centre Groningen, Department of Psychiatry, Groningen, The Netherlands

**Keywords:** antipsychotic discontinuation, antipsychotic medication, dopamine, First-episode psychosis, positron emission tomography, psychotic relapse

## Abstract

**Background:**

After remission of a first-episode psychosis (FEP), antipsychotic discontinuation is associated with an increased risk of relapse compared to maintenance treatment. We studied short and longer-term effects of discontinuation of D_2_ receptor (D_2_R) antagonist and partial agonist antipsychotics on striatal dopamine D_2/3_R availability in FEP patients.

**Methods:**

Remitted FEP patients underwent two [^11^C]raclopride PET scans to measure striatal D_2/3_R availability: 1 week after antipsychotic discontinuation (n = 16 antagonist users, n = 6 partial agonist users) and after being medication free for 6–8 weeks (n = 8 antagonist users, n = 5 partial agonist users). Fifteen matched healthy controls were scanned once. Psychotic relapse was monitored up to 12 months after discontinuation.

**Results:**

One week after discontinuation, D_2_R antagonist discontinuers showed higher striatal binding potential (BP_ND_) than partial D_2_R agonist discontinuers (*p* < 0.001, CI = 0.749 to 1.681) and controls (*p* = 0.045, CI = 0.008 to 0.708), while partial agonist discontinuers had significantly lower BP_ND_ than controls (*p* = 0.001, CI = -1.326 to -0.386). 6-8 weeks after discontinuation, former antagonist users showed similar BP_ND_ to controls (*p* > 0.25), whereas former partial agonist users had higher BP_ND_ than controls (*p* = 0.027, CI = 0.069 to 1.085). Participants who discontinued antagonists relapsed more often (81%) than those who discontinued partial agonists (17%)(χ^2^ = 5.32, *p* = 0.021).

**Conclusions:**

Discontinuation of partial D_2_R agonists may affect D_2/3_R availability differently than discontinuation of antagonists, which might explain the greater relapse risk after tapering antagonists than partial agonist antipsychotics.

## Introduction

Antipsychotic drugs effectively reduce psychotic symptoms and prevent relapse (Efthimiou et al., 2024; Højlund, Kemp, Haddad, Neill, & Correll, 2021; Leucht et al., 2021, 2012). These effects are thought to result from reduced striatal dopamine signalling (Kaar, Natesan, McCutcheon, & Howes, 2020; Strange, 2008). Antipsychotic drugs can be divided into D_2_ receptor (D_2_R) antagonists (e.g. olanzapine, haloperidol, risperidone), which decrease dopamine signalling by antagonizing dopamine D_2_R, and partial D_2_R agonists (e.g. aripiprazole, brexpiprazole), which stimulate D_2_R but to a lower extent than endogenous dopamine (Burris et al., 2002; Gründer, Kungel, Ebrecht, Göröcs, & Modell, 2006; Kinghorn & McEvoy, 2005).

Long-term exposure to antipsychotic treatment has been shown to alter pre- and postsynaptic dopamine neurotransmission and has been implied to upregulate D_2_R (Chouinard et al., 2017). This phenomenon has been reported for D_2_R antagonists, but little is known regarding the effects of partial agonists. The dopamine supersensitivity hypothesis states that long-term striatal D_2_R blockade by antipsychotics results in a compensatory upregulation of D_2_R expression and/or increase in the fraction of D_2_ receptors in the high-affinity state (Graff-Guerrero et al., 2009). Antipsychotic dose reduction or discontinuation after D_2_R upregulation is hypothesized to increase dopamine-mediated signalling, followed by a worsening of psychotic symptoms, called ‘supersensitivity psychosis’ (Servonnet & Samaha, 2020; Yin, Barr, Ramos-Miguel, & Procyshyn, 2017). Antipsychotic-induced D_2_R upregulation may therefore increase the risk for relapse and might contribute to the high relapse rates after antipsychotic discontinuation (Di Capite, Upthegrove, & Mallikarjun, 2018; Kishi et al., 2019; Leucht et al., 2012; Thompson et al., 2018; Zipursky, Menezes, & Streiner, 2014). Yet, it remains unknown whether such a D_2_R upregulation occurs directly after tapering and whether it is observed in both antagonist and partial agonist users.

Evidence for antipsychotic-induced D_2_R upregulation in humans is sparse and inconclusive. In a meta-analysis of human PET studies, a minor increase in D_2/3_R availability was observed in schizophrenia patients who had previously used antipsychotics, which was absent in drug-naïve patients (Howes et al., 2012). A PET study including nine patients with schizophrenia showed a 30% increase in D_2/3_R availability 14 days after discontinuation of moderate to high doses of haloperidol, perphenazine, risperidone, or olanzapine compared to eight antipsychotic-naïve patients (Silvestri et al., 2000). In contrast, no differences in striatal D_2/3_R availability were found between 14 controls and 25 FEP patients 7 weeks after discontinuation of aripiprazole, amisulpride, blonanserin, olanzapine, paliperidone, quetiapine, or risperidone (S. Kim et al., 2021).

For partial D_2_R agonists, this mechanism of D_2_R upregulation may not take place, as dopamine transmission is less affected compared to D_2_R antagonistic drugs. Animal studies indicate that partial D_2_R agonists do not cause dopamine supersensitivity (Chouinard et al., 2017). Rat studies showed that exposure to aripiprazole or brexpiprazole, in contrast to haloperidol, did not relate to increased D_2_R density (Amada et al., 2019; Tadokoro et al., 2012), which led to the suggestion that partial D_2_R agonists may prevent the development of dopamine supersensitivity (Tadokoro et al., 2012). Recently, high D_2_R affinity antagonist antipsychotics have been associated with a greater risk of relapse than low-affinity antagonists and partial agonist antipsychotics (Gangadin et al., 2025). This effect could result from stronger upregulation with high-affinity D_2_R antagonists. To better predict relapse risk and enable safe tapering of antipsychotic medication, a better understanding of neurobiological aspects of relapse risk after discontinuation is needed.

The present study examines the longitudinal effects of discontinuing partial D_2_R agonists and D_2_R antagonists at two timepoints: 1 week and 6–8 weeks after discontinuation, as dopamine supersensitivity may be a time-dependent process. D_2/3_R availability in striatum was measured with [^11^C]raclopride PET, using the non-displaceable binding potential (BP_ND_) as outcome parameter in 22 symptomatic remitted FEP patients and 15 matched controls for comparison. We hypothesized that discontinuation of D_2_R antagonists would result in higher D_2/3_R availability than partial D_2_R agonist discontinuation and controls after 1 week, with a normalization of D_2_R availability after 6–8 weeks. For partial D_2_R agonists, we did not expect any short or longer-term differences in D_2/3_R BP_ND_ compared to controls.

## Methods and Materials

### Participants

Patients were recruited from the HAMLETT trial (Handling Antipsychotic Medication: Long-term Evaluation of Targeted Treatment), a multicentre, single-blind randomized controlled trial in the Netherlands on antipsychotic medication reduction/discontinuation (Begemann et al., 2020) Participation in the present PET study was optional for those who completely discontinued their antipsychotic treatment. For the HAMLETT trial, participants were included if they were aged 16 to 60 years; used antipsychotic medication; had achieved symptomatic remission according to the treating psychiatrist for 3–6 months; had a DSM-5 or ICD-10 diagnosis of first episode of schizophrenia, schizoaffective disorder, schizophreniform disorder, brief psychotic disorder, delusional disorder, or unspecified schizophrenia spectrum and other psychotic disorder. Patients were excluded if they had exhibited dangerous or harmful behaviour during FEP. For the PET study, patients were excluded in case of a neurological disorder, substance dependency, and pregnancy.

Male healthy controls were scanned in a prior study (NL48500.042.14), and female healthy controls were recruited via advertisement. Control subjects had to be aged 18–60 years and were excluded from the study in case of a neurological disorder, substance dependency, pregnancy, MRI incompatible implants, history of psychiatric disorders or psychotropic medication use. This group was matched for age and sex to the patient group. All participants provided written informed consent after oral and written explanation of the study. Ethical approval was obtained from the research and ethics committee of the University Medical Center Groningen (NL64040.042.17 and NL48500.042.14).

### Study design

FEP patients who discontinued antipsychotic medication after a gradual tapering trajectory (tapering procedure and schedules are described in Supplementary Material 1) underwent two [^11^C]raclopride PET scans: 1 week and 6–8 weeks after discontinuation. Patients who relapsed or restarted antipsychotic medication after the first and before the second PET scan did not undergo the follow-up scan (n = 1 partial agonist user, n = 5 D_2_R antagonist users). Two antagonist discontinuers declined to participate in the follow-up scan. Blood samples were collected prior to the PET scan to determine antipsychotic concentrations. PET data of one follow-up scan of a participant with detectable antipsychotic serum concentrations (8 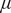 g/L olanzapine) were excluded from the analyses because the participant had restarted antipsychotic medication without notification. Participants were followed up for 12 months to assess relapse. Relapse was defined as hospitalization due to exacerbation of psychotic symptoms, judgement by the treating clinician, or reinstatement of antipsychotic medication. Medication use before and after initial antipsychotic discontinuation was derived from clinician reports and dispensation data from the Foundation for Pharmaceutical Statistics, from which olanzapine equivalents were calculated (Leucht et al., 2020; Leucht, Samara, Heres, & Davis, 2016). Antipsychotic drugs were classified into antagonists (i.e. olanzapine, risperidone, amisulpride) or partial D_2_R agonists (aripiprazole). Antipsychotic concentrations in plasma collected on the day of the first PET scan were not detectable, except in two patients who had discontinued aripiprazole and showed concentrations just above the lower limit of quantitation (12 and 13 μg/L; LLOQ 10 μg/L). Data from these patients were included in the analyses. Data from one person who used dexamphetamine was excluded from the analyses as dexamphetamine administration has been shown to lower D_2/3_R BP_ND_ (Weinstein et al., 2018). Supplementary Material 2 displays a flowchart of participants in the study. Prior to antipsychotic tapering, demographic characteristics and substance use were measured with the CASH (Andreassen, Flaum, & Arndt, 1992), psychotic illness severity was measured with the Positive and Negative Syndrome Scale (Kay, Fiszbein, & Opler, 1987), and global functioning was measured with the GAF (Jones, Thornicroft, Coffey, & Dunn, 1995).

### Imaging acquisition and kinetic analysis for [^**11**^**C]raclopride**

[^11^C]raclopride PET scans were performed with a Biograph mCT40 PET/CT camera (Siemens Medical Solutions, Knoxville, Tennessee, USA). The head of participants was fixed in a headrest to prevent motion artifacts and placed in the centre of the field of view of the PET/CT camera. Participants were required to refrain from alcohol for 24 hours, smoking for 12 hours, and eating for 4 hours before the PET scan, since alcohol, smoking and eating can stimulate the release of dopamine. A low-dose computed tomography (CT) scan was performed to correct for attenuation. Participants received a bolus injection of [^11^C]raclopride over a period of 1 minute, starting 10 seconds prior to the 60 minutes dynamic acquisition procedure. The mean (SD) injected dose of [^11^C]raclopride was 210 (25) MBq in controls and 205 (45) MBq in FEP patients at the first scan and 206 (50) MBq at the second scan. The injected dose did not differ between controls and FEP patients at the first (*t* = 0.42, *p* = 0.68) or second scan (*t* = 0.27, *p* = 0.79). Images were corrected for attenuation, scatter, and radioactive decay and iteratively reconstructed (3 iterations, 21 subsets) into 25 timeframes: frame 1–7: 10s, frame 8–9: 30s, frame 10–12: 60s, frame 13–14: 120s, frame 15–16: 180s, frame 17–18: 180s, frame 19–23: 300s, and frame 24–25: 600s.

On the same day as the first PET scan, an anatomic 1mm isotropic 3D T1 MRI scan was conducted with a 3 Tesla Magnetom Prisma MRI system (Siemens Medical Solutions USA, Inc). The average of all frames of the PET scan was used to coregister the PET scan to the individual T1 MRI scan and spatially normalized to the Montreal Neurological Institute (MNI) template, using PMOD (version 4.1, PMOD Technologies Ltd, Zürich, Switzerland). In case of missing MRI scans (n = 4), the R1 map of the PET images, obtained with the simplified reference tissue model (SRTM) (Lammertsma & Hume, 1996), was used as anatomical input for normalization. The Hammers maximum probability atlas (Hammers N3083 1MM) (Hammers et al., 2003) was used to define regions-of-interest for the cerebellum, putamen, caudate nucleus, nucleus accumbens and the striatum (i.e. caudate nucleus, nucleus accumbens, and putamen combined). Time activity curves were computed for all these brain areas. For each scan, the dopamine D_2/3_R BP_ND_ in striatal brain regions was calculated with SRTM2 (Wu & Carson, 2002) using the cerebellum as a reference region. The median k2’ of the entire cortex was determined with SRTM and used as input for SRTM2.

### Statistical analyses

A linear mixed effects model (LMEM) was applied to test whether the mean [^11^C]raclopride BP_ND_ differed between D_2_R antagonist users and partial D_2_R agonist users 1 week and 6–8 weeks after discontinuation, and controls (Supplementary Material 3). The BP_ND_ was used as dependent variable, with random intercepts for participants and fixed effects for time (first or second scan), the categorical variable group including D_2_R antagonist users, partial agonist users, and controls, and the interaction between time and group (Bates, D., Maechler, M., Bolker, B., & Walker, 2015). Similarly, LMEM with fixed effect for group, including relapse, no relapse, and controls, was used to test whether BP_ND_ differed between participants with or without relapse and controls. Planned contrasts with LMEM estimated marginal means (Searle, Speed, & Milliken, 1980) tested BP_ND_ differences between the D_2_R antagonists, partial D_2_R agonists, and controls at the first and second scan, and the change over time for the D_2_R antagonist and partial D_2_R agonist group. Similarly, contrasts were used to compare BP_ND_ in participants with and without relapse, and controls at the first and second scan, and the change in BP_ND_ between the scans for the relapse and nonrelapse group. Sensitivity analyses include LMEM on only the putamen and the caudate nucleus (Supplementary Material 4 and 5). A χ^2^ test was performed to assess whether the incidence of relapse differed in D_2_R antagonist and partial agonist discontinuers. Statistical analyses were performed in R (version 4.3.2) via Rstudio (version 2023.12.1.402) (R Core Team, 2020).

## Results

### Demographic and clinical characteristics

The analyses include 22 FEP patients (n = 4, 18% females; n = 18, 82% males) who underwent a [^11^C]raclopride PET scan approximately one week after antipsychotic discontinuation. Sixteen participants (73%) discontinued D_2_R antagonist antipsychotics (e.g. olanzapine n = 8, amisulpride n = 2, quetiapine n = 2, haloperidol n = 2) and 6 participants (28%) discontinued the partial D_2_R agonist aripiprazole. The follow-up [^11^C]raclopride PET scan at 6–8 weeks was completed by eight former antagonist and five former partial agonist users. Fifteen controls (n = 4, 27% females; n = 11, 73% males) underwent a single [^11^C]raclopride PET scan. Table 1 shows the demographic and clinical characteristics of the participants.

**Table 1.** Sociodemographic and clinical characteristics of FEP patients and controls

	Former D_2_R antagonist users	Former partial agonist users	All FEP patients	Controls
	(N = 16)	(N = 6)	(N = 22)	(N = 15)
**Sex at birth,** n (%)				
Female	3 (19%)	1 (17%)	4 (18%)	4 (27%)
Male	13 (81%)	5 (83%)	18 (82%)	11 (73%)
**Age,** mean (SD)	28 (7)	25 (8)	27 (7)	30 (9)
**Years of education,** mean (SD)	14.1 (2.4)	13.8 (4.1)	14.0 (2.9)	14.5 (2.3)
**Diagnosis**				
295.90 Schizophrenia	5 (31%)	2 (33%)	7 (32%)	
295.70 Schizoaffective disorder	5 (31%)	1 (17%)	6 (27%)	
295.40 Schizophreniform disorder	5 (31%)	3 (50%)	8 (36%)	
295.8 brief psychotic disorder	1 (6%)		1 (5%)	
**Duration of FEP (days),** mean (SD)	185 (296)	288 (443)	213 (334)	
**PANSS,^a^** mean (SD)				
Total	42.3 (11.0)	38.7 (3.7)	41.3 (9.6)	
Positive	9.3 (2.1)	8.0 (1.6)	9.0 (2.0)	
Negative	11.1 (4.2)	10.8 (2.2)	11.1 (3.7)	
General	21.8 (5.8)	19.8 (2.7)	21.3 (5.1)	
**Global functioning,^b^** mean (SD)	66.5 (13.2)	64.3 (8.6)	65.9 (11.9)	
**Type of antipsychotic discontinued,** n(%)				
Aripiprazol		6 (100%)	6 (27%)	
Amisulpride	2 (13%)		2 (9%)	
Flupentixol	1 (6%)		1 (5%)	
Haloperidol	2 (13%)		2 (9%)	
Olanzapine	8 (50%)		8 (36%)	
Quetiapine	2 (13%)		2 (9%)	
Risperidone	1 (6%)		1 (5%)	
**Last antipsychotic dose prior to complete discontinuation,** **^c^** mean (SD)	1.6 (0.9)	4.4 (4.4)	2.3 (2.6)	
**Number of days between discontinuation and 1** ^**st**^ **PET scan,** mean (SD)	7 (5)	8 (7)	8 (5)	
**Relapse after discontinuation,** n(%)				
No relapse	3 (19%)	5 (83%)	8 (36%)	
Relapse	13 (81%)	1 (17%)	14 (64%)	

a Positive and Negative Syndrome Scale (PANSS), measured at 3-6 months after remission from FEP.

b Global Assessment of Functioning (GAF), measured at 3-6 months after remission from FEP.

c In olanzapine equivalents mg/day.

### Short-term effects of discontinuation of partial D_**2**_**R agonist and D**_**2**_**R antagonist antipsychotics**

The linear mixed effects model (LMEM) with the antagonist group as reference showed a significant effect for group (partial D_2_R agonist users: estimate = -1.21, CI = -1.68 to -0.75, *p* < 0.001; control group: estimate = -0.36, CI = -0.71 to -0.01, *p* < 0.001) and the interaction between time and group (estimate = 1.54, CI = 0.84 to 2.24, *p* < 0.0001 for partial agonist by 2-month follow-up interaction) (Supplementary Material 3).

**Figure 1.**
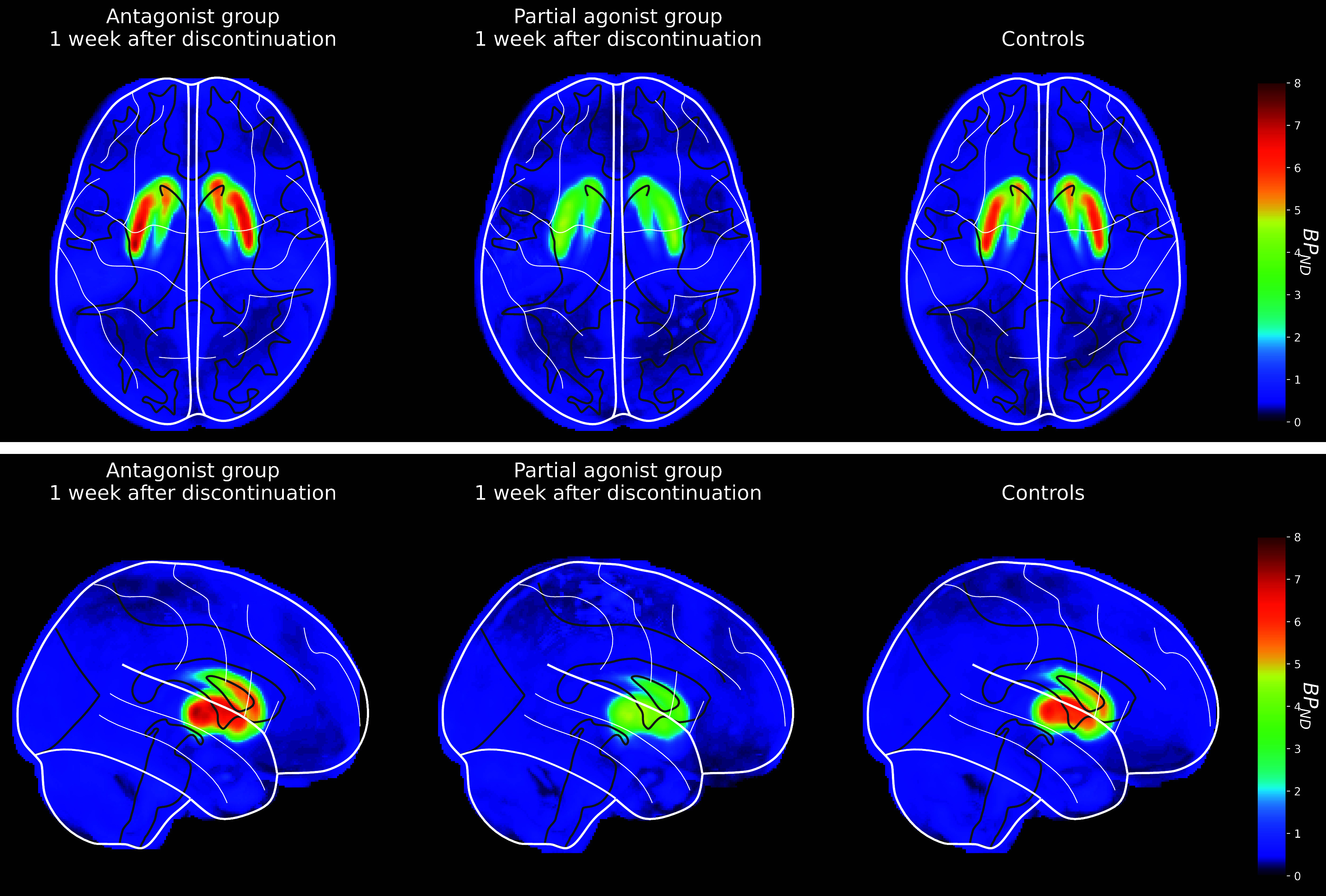
Averaged and normalized [^11^C]raclopride PET BP_ND_ maps showing D_2/3_R availability in the striatum for FEP patients 1 week after discontinuation of treatment with antagonist (left) or partial agonist (middle) antipsychotics and controls (right). Images are shown in axial (a) and sagittal (b) views.

One week after antipsychotic discontinuation, the BP_ND_ in striatum was significantly higher in FEP patients who discontinued D_2_R antagonists (mean 3.98, SD 0.48) compared to partial D_2_R agonist (mean 2.76, SD 0.66; *t* = 5.250, *p* < 0.001, CI = 0.749 to 1.681) and controls (mean 3.62, SD 0.43; *t* = 2.063, *p* = 0.045, CI = 0.008 to 0.708) (Figure 1). The striatal BP_ND_ was lower in participants who had discontinued partial D_2_R agonists in the past week than controls (t = -3.668, p = 0.001, CI = -1.326 to -0.386).

### Longer-term effects of discontinuation of partial D_**2**_R agonist and D_2_R antagonist antipsychotics

**Figure 2.**
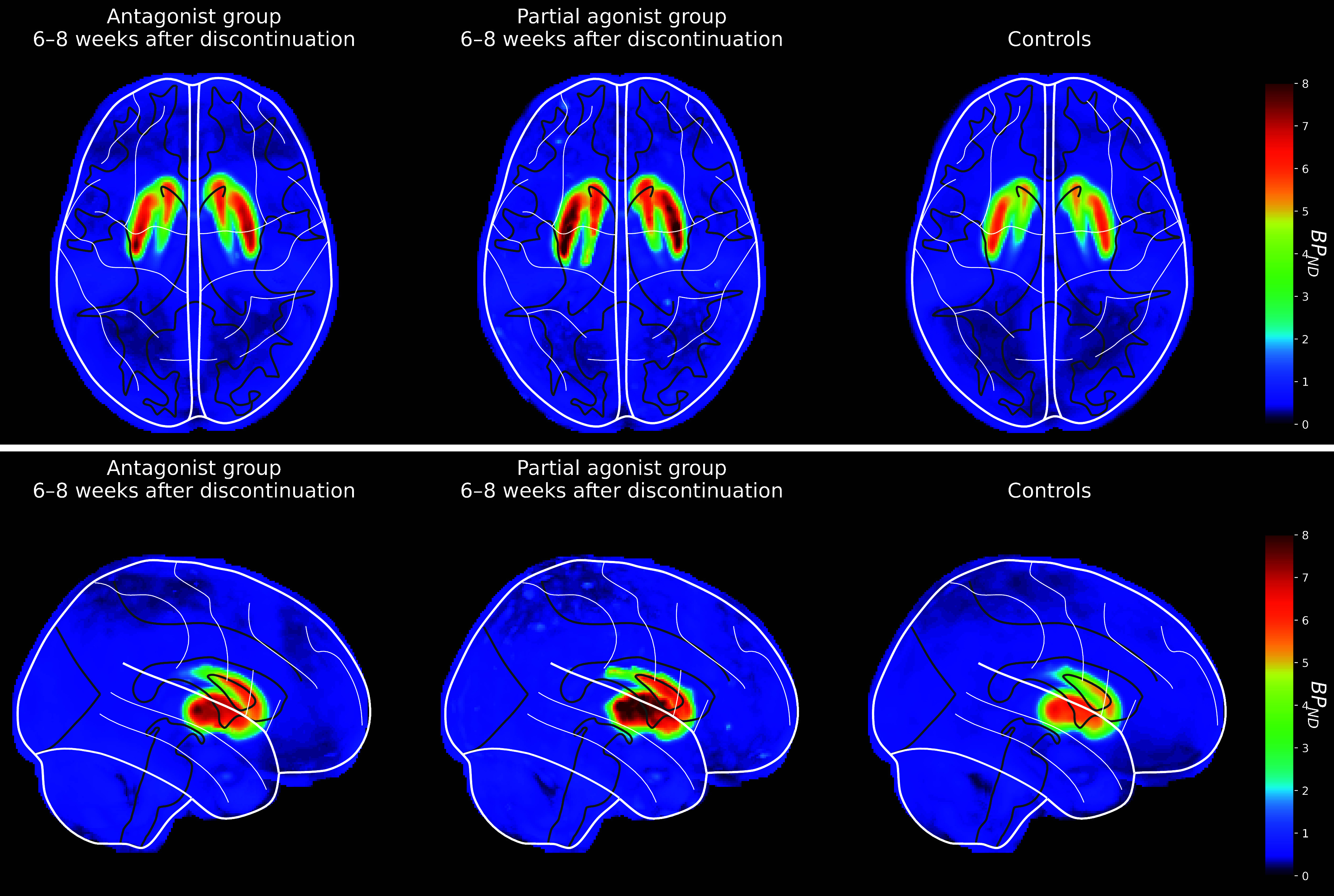
Averaged and normalized [^11^C]raclopride PET BP_ND_ maps showing D_2/3_R availability in striatum for FEP patients 6-8 weeks after discontinuation with antagonist antipsychotics (left) or partial agonist antipsychotics (middle) and controls (right). Images are shown in axial (a) and sagittal (b) views.

At 6-8 weeks follow-up, there was no difference in BP_ND_ between participants who had discontinued D_2_R antagonists and controls (*t* = 1.166, *p* = 0.250, CI = -0.184 to 0.691). Striatal BP_ND_ at 6-8 weeks follow-up was higher in former partial agonist users (mean 4.21, SD 0.45) than controls (mean 3.62, SD 0.43; *t* = 2.289, *p* = 0.027, CI = 0.069 to 1.085), but did not differ between former partial D_2_R agonist users and former D_2_R antagonist users (mean 3.88, SD 0.44; *t* = 1.147, *p =* 0.257, CI = -0.245 to 0.893) (Figure 2). In partial agonist discontinuers, there was a significant increase in striatal BP_ND_ over time (*t =* -5.063, *p* < 0.001, CI = -2.034 to -0.832), while the BP_ND_ did not differ significantly over time in former D_2_R antagonist users (*t =* 0.502, *p =* 0.621, CI = -0.329 to 0.539).

### Psychotic relapse after antipsychotic discontinuation

**Figure 3.**
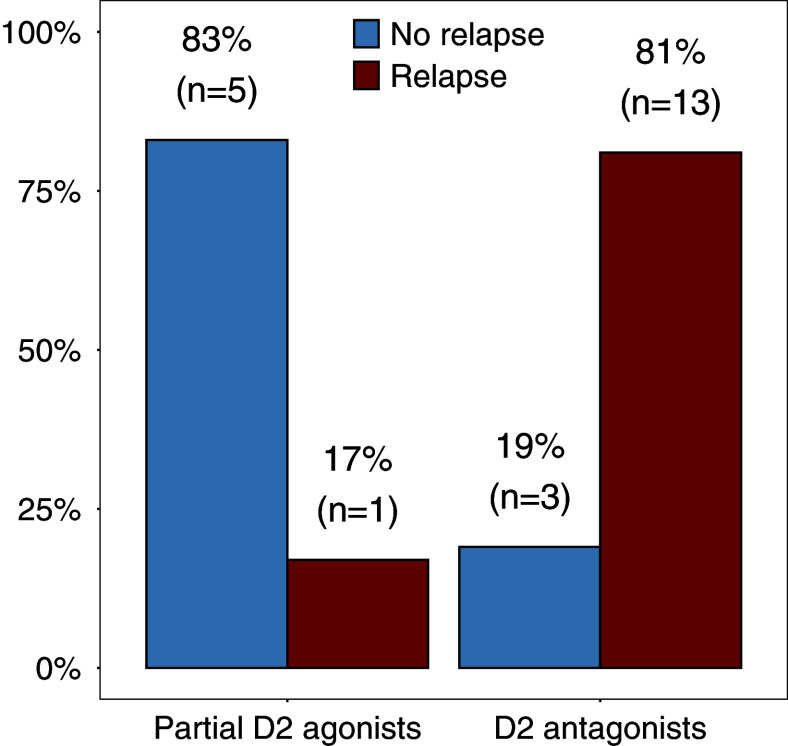
Frequency of annual psychotic relapse following antipsychotic discontinuation in FEP patients who stopped D_2_ partial D_2_R agonist (n = 6) and D_2_R antagonist (n = 16) antipsychotics.

Fourteen FEP participants (64%) experienced relapse within the 12 months after antipsychotic discontinuation, while eight participants (36%) did not experience relapse. Five former D_2_R antagonist users and one former partial D_2_R agonist user experienced a relapse or symptom return and restarted antipsychotic treatment before the second scan at two months follow-up. Relapses occurred more often in participants who discontinued D_2_R antagonists than partial D_2_R agonists (χ^2^ = 5.32, *p* = 0.021) (Figure 3).

LMEM showed an interaction between time and group (estimate = 1.01, CI = 0.17 to 1.85, *p* = 0.020 for no relapse by 2-month follow-up interaction). The BP_ND_ in striatum one week after discontinuation showed a trend towards lower values in patients who remained stable (mean: 3.35, SD 0.89) than in those who relapsed (mean 3.82, SD 0.65) (*t* = -1.789, *p* = 0.080, CI = -0.983 to 0.058). There was no significant difference in BP_ND_ between controls and participants with (*t* = -0.901, *p* = 0.372, CI = -0.632 to 0.241) or without relapse (*t* = 1.046, *p* = 0.301, CI = -0.247 to 0.781) one week after discontinuation.

After being without medication for 2 months, the BP_ND_ in striatum was significantly higher in participants without relapse (mean 4.21, SD 0.38) than in controls (*t* = 2.324, *p* = 0.025, CI = 0.079 to 1.107). The BP_ND_ after 2 months did not differ between patients with later relapse (mean 3.67, SD 0.38) compared to those who remained stable (*t* = -1.583, *p* = 0.120, CI = -1.238 to 0.148), or controls (t = 0.154, p = 0.878, CI = -0.584 to 0.681). In FEP patients who remained stable, BP_ND_ showed a significant increase over time (+26%; *t* = -2.990, *p* = 0.009, CI = -1.475 to -0.245), while BP_ND_ did not change over time in those who relapsed within a year (-4%; *t* = 0.467, *p* = 0.644, CI = -0.497 to 0.791).

## Discussion

The present study is the first to test short and longer-term molecular effects of discontinuing D_2_R antagonist and partial D_2_R agonists antipsychotics on D_2/3_R availability in 22 remitted FEP patients. One week after discontinuation, D_2_R antagonist discontinuers showed increased D_2/3_R availability compared to controls, while partial agonist users showed decreased D_2/3_R availability 1 week after discontinuation. After 2 months without medication, former antagonist users showed similar D_2/3_R availability to controls, while former partial agonist users showed increased D_2/3_R availability.

### Short-term effects of discontinuation of D_2_R antagonist and partial D_2_R agonist antipsychotics

Our short-term findings support the dopamine supersensitivity hypothesis for antagonist antipsychotics and are in line with previous studies showing D_2/3_R upregulation after antipsychotic use in schizophrenia (Howes et al., 2012). These results may also provide a neurobiological understanding of the particularly high relapse risk after tapering high D_2_R affinity antipsychotics in a previous study with FEP patients, from which the participants of the present study were a subset (Gangadin et al., 2025). Exposure to high D_2_R affinity antipsychotics could lead to a stronger D_2_R blockade and hence greater D_2_R upregulation, which could increase relapse risk after antipsychotic dose-reduction/discontinuation.

In partial D_2_R agonist discontinuers, D_2/3_R availability was lower compared to both antagonist discontinuers and healthy controls one week after discontinuation. As aripiprazole is a partial agonist, its use may have caused a downregulation of D_2_R as compared to controls. However, the lower availability could also reflect a transient increase in dopamine synthesis in the patients who discontinued aripiprazole. Given aripiprazole’s long half-life of 75 hours (Casey & Canal, 2017), a minimal trace of the drug may still have been present 1 week after discontinuation during the first PET scan, potentially occupying some D_2_R. Although most partial agonist users had undetectable blood levels at scanning (mean 8 days post-discontinuation), two participants had low but detectable levels. Although residual drug traces may have had some effect, the robust differences we observed 1 week after antagonist and partial agonist cessation are too large to be solely the result of residual aripiprazole in the brain of former agonist users.

### Longer-term effects of antipsychotic discontinuation and the risk of relapse

At 2 months follow-up, D_2/3_R availability in antagonist discontinuers was no longer significantly different from controls. Former users or the partial agonist aripiprazole showed a significant increase in D_2/3_R availability over the course of 2 months after discontinuation, to eventually higher D_2/3_R availability at 6-8 weeks follow-up than controls.

Consistent with findings from the larger group of which the participants in the present study were part of, aripiprazole users less frequently experienced relapse after discontinuation than high-affinity antagonist antipsychotics (Gangadin et al., 2025). Twelve months after discontinuation, 83% of former partial D_2_R agonist users remained relapse-free compared to only 17% of the antagonist group, despite similar baseline characteristics.

Participants without relapse showed a significant increase in D_2/3_R availability over time, resulting in higher D_2/3_R availability after two medication-free months in comparison to controls. As psychotic relapse has been associated with increased dopamine synthesis (Abi-Dargham et al., 2004, 2000; Cheng et al., 2020; Howes et al., 2011, 2012; S. Kim et al., 2021; Weidenauer et al., 2020), a tentative explanation to reconcile the higher D_2/3_R availability with lower propensity for relapse is lower levels of endogenous dopamine in former partial agonist users and in those who remained relapse free. Alternatively, one may argue that both FEP patients who discontinued D_2/3_R antagonists as well as partial agonists eventually show greater D_2/3_R availability than controls. This effect, however, took longer to manifest in former partial agonist users, and the slower pace of increase in D_2/3_R availability may be protective against relapse.

### Strengths and limitations

An important strength of this study is that we assessed both short and longer-term effects of antipsychotic discontinuation on D_2/3_R availability. Also, the differentiation between D_2_R antagonistic and partial agonistic antipsychotics is an important aspect, as these types of drugs may affect the dopamine system in different ways. Another strength is the focus on FEP patients as the study population, combined with the detailed clinical description of the participants prior to discontinuation.

A key limitation is the limited sample size, especially at the longer follow-up scan. From the 16 patients who discontinued, 6 restarted antipsychotics within 2 months, illustrating their vulnerability to relapse. Furthermore, a hurdle for the interpretation of results is the inability to separate the effects of different molecular processes affecting the [^11^C]raclopride BP_ND_ after antipsychotic discontinuation. The [^11^C]raclopride BP_ND_ is sensitive to the level of endogenous dopamine (Ginovart, 2005) and the observed dorsal striatal binding potential results from both the number of D_2_R—and to a lesser extent D_3_R—, as well as from endogenous dopamine synthesis, release, and clearance (McCormick, Kapur, Seeman, & Wilson, 2008). Dopamine neurotransmission is a complex process, and changes in dopamine production, release or transport also affect the BP_ND_ of the post-synaptic tracer. Additionally, we cannot rule out the minimal presence of antipsychotic drugs during the first scan, especially for those who discontinued aripiprazole. We could have standardized dopamine synthesis capacity after antipsychotic discontinuation by using the amphetamine challenge paradigm (Laruelle et al., 1996, 1995; Volkow et al., 1994). However, considering that participants were vulnerable for relapse, we did not deem this experimental paradigm ethically justified. Similarly, the dopamine depletion paradigm (Laruelle et al., 1997), which provides an estimate of D_2_R occupancy by endogenous dopamine and includes two PET scans within a 48-hour interval and alpha-methyl-para-tyrosine (AMPT) administration every 6 hours for 2-3 days, was considered too demanding for our delicate group. As participants were not randomized by the type of antipsychotic drug, there is a risk of bias by indication, even though baseline demographic and clinical characteristics were very similar between the D_2_R antagonist partial agonist groups. Another limitation is the relatively small sample size at follow-up, as patients who relapsed quickly after discontinuation had to be excluded, which may have limited our power in some of the analyses.

## Conclusions

The present study is the first to assess the short and longer-term effects of the discontinuation of D_2_R antagonist and a partial D_2_R agonist antipsychotic on D_2/3_R availability in remitted FEP patients. D_2_R antagonist discontinuers showed increased D_2/3_R availability one week after cessation, and 81% experienced a relapse within 12 months. These findings are in line with the dopamine supersensitivity hypothesis. In contrast, partial agonist users had a much lower relapse incidence of 17% and showed decreased D_2/3_R availability shortly after discontinuation and an increased D_2/3_R availability after 2 months. These findings indicate that discontinuation of partial D_2_R agonists may affect D_2/3_R differently than D_2_R antagonist antipsychotics, which may be related to the lower relapse rates associated with partial D_2_R agonists. If replicated, current findings point to a preference for treatment with partial agonists.

## Supporting information

de Beer et al. supplementary material de Beer et al. supplementary material

## Supplementary material

The supplementary material for this article can be found at http://doi.org/10.1017/S003329172510161X.

## References

[r1] Abi-Dargham, A., Kegeles, L. S., Zea-Ponce, Y., Mawlawi, O., Martinez, D., Mitropoulou, V., & Siever, L. J. (2004). Striatal amphetamine-induced dopamine release in patients with schizotypal personality disorder studied with single photon emission computed tomography and [123I]iodobenzamide. Biological Psychiatry, 55(10), 1001–1006. 10.1016/j.biopsych.2004.01.018. 15121484

[r2] Abi-Dargham, A., Rodenhiser, J., Printz, D., Zea-Ponce, Y., Gil, R., Kegeles, L. S., & Laruelle, M. (2000). Increased baseline occupancy of D _2_ receptors by dopamine in schizophrenia. Proceedings of the National Academy of Sciences, 97(14), 8104–8109. 10.1073/pnas.97.14.8104. PMC16677 10884434

[r3] Amada, N., Akazawa, H., Ohgi, Y., Maeda, K., Sugino, H., Kurahashi, N., & Futamura, T. (2019). Brexpiprazole has a low risk of dopamine D _2_ receptor sensitization and inhibits rebound phenomena related to D _2_ and serotonin 5-HT _2A_ receptors in rats. Neuropsychopharmacology Reports, 39(4), 279–288. 10.1002/npr2.12076. 31487433 PMC7292306

[r4] Andreassen, N., Flaum, M., & Arndt, S. (1992). The comprehensive assessment of symptoms and history (CASH) an instrument for assessing diagnosis and psychopathology. Archives of General Psychiatry, 49(8), 615–623. 10.1001/archpsyc.1992.01820080023004. 1637251

[r5] Bates, D., Maechler, M., Bolker, B., & Walker, S. (2015). Fitting linear mixed-effects models using lme4. Journal of Statistical Software, 67(1), 1–48.

[r6] Begemann, M. J. H., Thompson, I. A., Veling, W., Gangadin, S. S., Geraets, C. N. W., Van’T Hag, E., … Sommer, I. E. C. (2020). To continue or not to continue? Antipsychotic medication maintenance versus dose-reduction/discontinuation in first episode psychosis: HAMLETT, a pragmatic multicenter single-blind randomized controlled trial. Trials, 21(1). 10.1186/s13063-019-3822-5 PMC7006112 32033579

[r7] Burris, K. D., Molski, T. F., Xu, C., Ryan, E., Tottori, K., Kikuchi, T., & Molinoff, P. B. (2002). Aripiprazole, a novel antipsychotic, is a high-affinity partial agonist at human dopamine D2 receptors. Journal of Pharmacology and Experimental Therapeutics, 302(1), 381–389. 10.1124/jpet.102.033175. 12065741

[r8] Casey, A. B., & Canal, C. E. (2017). Classics in chemical neuroscience: Aripiprazole. ACS Chemical Neuroscience, 8(6), 1135–1146. 10.1021/acschemneuro.7b00087. 28368577 PMC5495458

[r9] Cheng, P. W. C., Chang, W. C., Lo, G. G., Chan, K. W. S., Lee, H. M. E., Hui, L. M. C., & Howes, O. D. (2020). The role of dopamine dysregulation and evidence for the transdiagnostic nature of elevated dopamine synthesis in psychosis: A positron emission tomography (PET) study comparing schizophrenia, delusional disorder, and other psychotic disorders. Neuropsychopharmacology, 45(11), 1870–1876. 10.1038/s41386-020-0740-x. 32612207 PMC7608388

[r10] Chouinard, G., Samaha, A. N., Chouinard, V. A., Peretti, C. S., Kanahara, N., Takase, M., & Iyo, M. (2017). Antipsychotic-induced dopamine Supersensitivity psychosis: Pharmacology, criteria, and therapy. Psychotherapy and Psychosomatics, 86(4), 189–219. 10.1159/000477313. 28647739

[r11] Di Capite, S., Upthegrove, R., & Mallikarjun, P. (2018). The relapse rate and predictors of relapse in patients with first-episode psychosis following discontinuation of antipsychotic medication. Early Intervention in Psychiatry, 12(5), 893–899. 10.1111/eip.12385. 27734591

[r12] Efthimiou, O., Taipale, H., Radua, J., Schneider-Thoma, J., Pinzón-Espinosa, J., Ortuño, M., & Luykx, J. J. (2024). Efficacy and effectiveness of antipsychotics in schizophrenia: Network meta-analyses combining evidence from randomised controlled trials and real-world data. The Lancet Psychiatry, 11(2), 102–111. 10.1016/S2215-0366(23)00366-8. 38215784

[r13] Gangadin, S. S., de Beer, F., Wijnen, B., Begemann, M., van Beveren, N., Boonstra, N., & Sommer, I. E. C. (2025). Risk of relapse during tapering of antipsychotic medication after a first psychotic episode: Association with D2 receptor affinity but not with tapering speed. World Psychiatry, 24(2), 240–249. 10.1002/wps.21315. 40371797 PMC12079330

[r14] Ginovart, N. (2005). Imaging the dopamine system with in vivo [11C]raclopride displacement studies: Understanding the true mechanism. Molecular Imaging and Biology, 7(1), 45–52. 10.1007/s11307-005-0932-0. 15912275

[r15] Graff-Guerrero, A., Mamo, D., Shammi, C. M., Mizrahi, R., Marcon, H., Barsoum, P., … Kapur, S. (2009). The effect of antipsychotics on the high-affinity state of D2 and D3 receptors: A positron emission tomography study with [11C]-(+)-PHNO. Archives of General Psychiatry, 66(6), 606–615. 10.1001/archgenpsychiatry.2009.43. 19487625

[r16] Gründer, G., Kungel, M., Ebrecht, M., Göröcs, T., & Modell, S. (2006). Aripiprazole: Pharmacodynamics of a dopamine partial agonist for the treatment of schizophrenia. Pharmacopsychiatry, 39 (SUPPL. 1). 10.1055/s-2006-931485. 16508892

[r17] Hammers, A., Allom, R., Koepp, M. J., Free, S. L., Myers, R., Lemieux, L., & Duncan, J. S. (2003). Three-dimensional maximum probability atlas of the human brain, with particular reference to the temporal lobe. Human Brain Mapping, 19(4), 224–247. 10.1002/hbm.10123. 12874777 PMC6871794

[r18] Højlund, M., Kemp, A. F., Haddad, P. M., Neill, J. C., & Correll, C. U. (2021). Standard versus reduced dose of antipsychotics for relapse prevention in multi-episode schizophrenia: A systematic review and meta-analysis of randomised controlled trials. The Lancet Psychiatry, 8(6), 471–486. 10.1016/S2215-0366(21)00078-X. 34023019

[r19] Howes, O. D., Bose, S. K., Turkheimer, F., Valli, I., Egerton, A., Valmaggia, L. R., & McGuire, P. (2011). Dopamine synthesis capacity before onset of psychosis: A prospective [ ^18^ F]-DOPA PET imaging study. American Journal of Psychiatry, 168(12), 1311–1317. 10.1176/appi.ajp.2011.11010160. 21768612 PMC3682447

[r20] Howes, O. D., Kambeitz, J., Kim, E., Stahl, D., Slifstein, M., Abi-Dargham, A., & Kapur, S. (2012). The nature of dopamine dysfunction in schizophrenia and what this means for treatment: Meta-analysisof imaging studies. Archives of General Psychiatry, 69(8), 776–786. 10.1001/archgenpsychiatry.2012.169. 22474070 PMC3730746

[r21] Jones, S. H., Thornicroft, G., Coffey, M., & Dunn, G. (1995). A brief mental health outcome scale-reliability and validity of the global assessment of functioning (GAF). The British Journal of Psychiatry : The Journal of Mental Science, 166(5), 654–659. 10.1192/bjp.166.5.654. 7620753

[r22] Kaar, S. J., Natesan, S., McCutcheon, R., & Howes, O. D. (2020). Antipsychotics: Mechanisms underlying clinical response and side-effects and novel treatment approaches based on pathophysiology. Neuropharmacology, 172, 107704. 10.1016/j.neuropharm.2019.107704. 31299229

[r23] Kay, S. R., Fiszbein, A., & Opler, L. A. (1987). The positive and negative syndrome scale (PANSS) for schizophrenia. Schizophrenia Bulletin, 13(2), 261–276. 10.1093/schbul/13.2.261. 3616518

[r25] Kim, S., Shin, S. H., Santangelo, B., Veronese, M., Kang, S. K., Lee, J. S., & Kim, E. (2021). Dopamine dysregulation in psychotic relapse after antipsychotic discontinuation: An [18F]DOPA and [11C]raclopride PET study in first-episode psychosis. Molecular Psychiatry, 26(7), 3476–3488. 10.1038/s41380-020-00879-0. 32929214

[r26] Kinghorn, W. A., & McEvoy, J. P. (2005). Aripiprazole: Pharmacology, efficacy, safety and tolerability. Expert Review of Neurotherapeutics, 5(3), 297–307. 10.1586/14737175.5.3.297. 15938662

[r27] Kishi, T., Ikuta, T., Matsui, Y., Inada, K., Matsuda, Y., Mishima, K., & Iwata, N. (2019). Effect of discontinuation *v.* maintenance of antipsychotic medication on relapse rates in patients with remitted/stable first-episode psychosis: A meta-analysis. Psychological Medicine, 49(5), 772–779. 10.1017/S0033291718001393. 29909790

[r28] Lammertsma, A. A., & Hume, S. P. (1996). Simplified reference tissue model for PET receptor studies. NeuroImage, 4(3), 153–158. 10.1006/nimg.1996.0066. 9345505

[r29] Laruelle, M., Abi-Dargham, A., van Dyck, C. H., Gil, R., D’Souza, C. D., Erdos, J., & Innis, R. B. (1996). Single photon emission computerized tomography imaging of amphetamine-induced dopamine release in drug-free schizophrenic subjects. Proceedings of the National Academy of Sciences, 93(17), 9235–9240. 10.1073/pnas.93.17.9235. PMC38625 8799184

[r30] Laruelle, M., Abi-Dargham, A., van Dyck, C. H., Rosenblatt, W., Zea-Ponce, Y., Zoghbi, S. S., & Kung, H. F. (1995). SPECT imaging of striatal dopamine release after amphetamine challenge. Journal of Nuclear Medicine: Official Publication, Society of Nuclear Medicine, 36(7), 1182–1190. 7790942

[r31] Laruelle, M., D’Souza, C. D., Baldwin, R. M., Abi-Dargham, A., Kanes, S. J., Fingado, C. L., & Innis, R. B. (1997). Imaging D2 receptor occupancy by endogenous dopamine in humans. Neuropsychopharmacology, 17(3), 162–174. 10.1016/S0893-133X(97)00043-2. 9272483

[r32] Leucht, S., Bauer, S., Siafis, S., Hamza, T., Wu, H., Schneider-Thoma, J., & Davis, J. M. (2021). Examination of dosing of antipsychotic drugs for relapse prevention in patients with stable schizophrenia. JAMA Psychiatry, 78(11), 1238. 10.1001/jamapsychiatry.2021.2130. PMC8374744 34406325

[r33] Leucht, S., Crippa, A., Siafis, S., Patel, M. X., Orsini, N., & Davis, J. M. (2020). Dose-response meta-analysis of antipsychotic drugs for acute schizophrenia. American Journal of Psychiatry, 177(4), 342–353. 10.1176/appi.ajp.2019.19010034. 31838873

[r34] Leucht, S., Samara, M., Heres, S., & Davis, J. M. (2016). Dose equivalents for antipsychotic drugs: The DDD method. Schizophrenia Bulletin, 42, S90–S94. 10.1093/schbul/sbv167. 27460622 PMC4960429

[r35] Leucht, S., Tardy, M., Komossa, K., Heres, S., Kissling, W., Salanti, G., & Davis, J. M. (2012). Antipsychotic drugs versus placebo for relapse prevention in schizophrenia: A systematic review and meta-analysis. The Lancet, 379(9831), 2063–2071. 10.1016/S0140-6736(12)60239-6. 22560607

[r36] McCormick, P. N., Kapur, S., Seeman, P., & Wilson, A. A. (2008). Dopamine D2 receptor radiotracers [11C](+)-PHNO and [3H]raclopride are indistinguishably inhibited by D2 agonists and antagonists ex vivo. Nuclear Medicine and Biology, 35(1), 11–17. 10.1016/j.nucmedbio.2007.08.005. 18158938

[r37] R Core Team. (2020). R: A language and environment for statistical computing. R foundation for statistical computing. Vienna, Australia: R Foundation for Statistical Computing. www.R-Project.org.

[r38] Searle, S. R., Speed, F. M., & Milliken, G. A. (1980). Population marginal means in the linear model: An alternative to least squares means. The American Statistician, 34(4), 216–221. 10.1080/00031305.1980.10483031.

[r39] Servonnet, A., & Samaha, A.-N. (2020). Antipsychotic-evoked dopamine supersensitivity. Neuropharmacology, 163, 107630. 10.1016/j.neuropharm.2019.05.007. 31077727

[r41] Silvestri, S., Seeman, M. V., Negrete, J.-C., Houle, S., Shammi, C. M., Remington, G. J., & Seeman, P. (2000). Increased dopamine D 2 receptor binding after long-term treatment with antipsychotics in humans: A clinical PET study. Psychopharmacology, 152(2), 174–180. 10.1007/s002130000532. 11057521

[r43] Strange, P. (2008). Antipsychotic drug action: Antagonism, inverse agonism or partial agonism. Trends in Pharmacological Sciences, 29(6), 314–321. 10.1016/j.tips.2008.03.009. 18471899

[r44] Tadokoro, S., Okamura, N., Sekine, Y., Kanahara, N., Hashimoto, K., & Iyo, M. (2012). Chronic treatment with aripiprazole prevents development of dopamine supersensitivity and potentially supersensitivity psychosis. Schizophrenia Bulletin, 38(5), 1012–1020. 10.1093/schbul/sbr006. 21402722 PMC3446226

[r46] Thompson, A., Winsper, C., Marwaha, S., Haynes, J., Alvarez-Jimenez, M., Hetrick, S., & Sullivan, S. A. (2018). Maintenance antipsychotic treatment versus discontinuation strategies following remission from first episode psychosis: Systematic review. BJPsych Open, 4(4), 215–225. 10.1192/bjo.2018.17. 29988997 PMC6034451

[r48] Volkow, N. D., Wang, G., Fowler, J. S., Logan, J., Schlyer, D., Hitzemann, R., & Wolf, A. P. (1994). Imaging endogenous dopamine competition with [ ^11^ C]raclopride in the human brain. Synapse, 16(4), 255–262. 10.1002/syn.890160402. 8059335

[r49] Weidenauer, A., Bauer, M., Sauerzopf, U., Bartova, L., Nics, L., Pfaff, S., & Willeit, M. (2020). On the relationship of first-episode psychosis to the amphetamine-sensitized state: A dopamine D2/3 receptor agonist radioligand study. Translational Psychiatry, 10(1), 2. 10.1038/s41398-019-0681-5. PMC7026156 32066718

[r50] Weinstein, J. J., van de Giessen, E., Rosengard, R. J., Xu, X., Ojeil, N., Brucato, G., & Abi-Dargham, A. (2018). PET imaging of dopamine-D2 receptor internalization in schizophrenia. Molecular Psychiatry. 10.1038/mp.2017.157. PMC5690884 28507321

[r51] Wu, Y., & Carson, R. E. (2002). Noise reduction in the simplified reference tissue model for Neuroreceptor functional imaging. Journal of Cerebral Blood Flow & Metabolism, 22(12), 1440–1452. 10.1097/01.WCB.0000033967.83623.34. 12468889

[r52] Yin, J., Barr, A. M., Ramos-Miguel, A., & Procyshyn, R. M. (2017). Antipsychotic induced dopamine Supersensitivity psychosis: A comprehensive review. Current Neuropharmacology, 15(1), 174–183. 10.2174/1570159x14666160606093602. 27264948 PMC5327459

[r53] Zipursky, R. B., Menezes, N. M., & Streiner, D. L. (2014). Risk of symptom recurrence with medication discontinuation in first-episode psychosis: A systematic review. Schizophrenia Research, 152(2–3), 408–414. 10.1016/j.schres.2013.08.001. 23972821

