# Supplementary material for: Dopamine D2/3R availability after discontinuation of antipsychotic treatment: a [11C]raclopride PET study in remitted first-episode psychosis patients
Source: Psychol Med. 2025 Sep 8;55:e264. doi: 10.1017/S003329172510161X (PMC13040595; doi:10.1017/S003329172510161X)
Supplement: de Beer et al. supplementary material [file S003329172510161Xsup001.docx]

**Supplementary Material**

**Supplementary Material 1. HAMLETT tapering procedure**

The HAMLETT tapering guide was provided to participants in the DR/D group by their treating physician during the first clinical consultation after randomization. The guide was provided in physical form, and also available online at [www.HAMLETT.nl](https://assets-eu-01.kc-usercontent.com/2e33a617-818c-01cf-b1cc-592ff1c94103/babca4a6-20c5-49ee-8543-0f9d5aae393a/Afbouwfolder_HAMLETT_november_2023.pdf). Key topics discussed included the benefits and risks of tapering, recommended tapering schedules, and the importance of having a close support person involved. Together with the participant, potential warning signs were documented. The same procedure was provided to the maintenance group when tapering was considered at a later timepoint (i.e. after 12 months).

*General information on tapering*

The tapering guide restates the goal and study procedure of the HAMLETT study and provides information on the risks and benefits of tapering. The guide repeatedly stresses the importance of good and timely communication with the treating physician during tapering. Participants were also informed about what to expect during tapering, including the time needed to readjust. Withdrawal symptoms can occur while tapering, which makes it important to taper slowly. Additionally, as the protective effects of antipsychotics diminish during tapering, the risk of rebound or relapse increases. To help prepare participants, information was provided on early warning signs.

*Early warning signs*

The early warning signs as described by Orygen [60] were translated into Dutch. Participants could indicate whether the early warning sign was present before or during their first psychotic episode. Participants were encouraged to also include the perspective of a close support person who was in frequent contact with them during the prodromal phase. Based on this information, an early warning action plan was devised in collaboration with the participant, a close support person and the treating physician. In addition, the participant could also mark down in the guide whether a specific early warning sign occurred during tapering. It was strongly emphasized that participants should contact their treating physicians if early warning signs occurred. The guide included a diary template for participants to record their daily dose and how they felt during the tapering period.

*Tapering schedules*

The tapering schedules started from the maximal recommended dose according to the Summary of Product Characteristics (SPCs) of the antipsychotics, to provide comprehensive information. In practice, remission was frequently reached on lower doses, and consequently, the tapering schedules lasted shorter than described on the next page. Following recommendations later published by Horowitz et al. (2021),we ensured that the final steps in the tapering schedules were as gradual as possible, considering the available dosages.

*Social support*
After signing informed consent, HAMLETT participants were asked to designate a specific close support person the role of a ‘tapering buddy’. This person served as a contact for both the study and clinical teams and was aware of when and how the participant would taper off medication. Participants received two copies of the tapering guide: one for themselves and one for their close support person. The guide also provided information for the tapering buddy on how to support the participant during tapering. For example, the guide facilitated monitoring of the early warning signs by the tapering buddy.

**HAMLETT Tapering schedules**

Table S1 Tapering schedules provided as guidelines for gradual discontinuation per antipsychotic drug based on regular available doses.

|  | Max. dose SPC | Available doses | Start tapering | After 2w | After 4w | After 6 w | After 8w | After 10w | After 12w | After 14w | After 16w | After 18w | After 20w | After 22w | After 24w | After 26w | After 28w | After 30w |
| --- | --- | --- | --- | --- | --- | --- | --- | --- | --- | --- | --- | --- | --- | --- | --- | --- | --- | --- |
| Risperidone | 10 | 0.5, 1, 2, 3, 4,6 | 10 | 8 | 6 | 5 | 4 | 3 | 2 | 1.5 | 1 | 0.5 | 0.5 | 0.25 | 0.25 | 0.25** | 0.25** | stop |
| Olanzapine | 20 | 2.5, 5, 10, 15, 20 | 20 | 17.5 | 15 | 12.5 | 10 | 7.5 | 5 | 5 | 2.5 | 2.5 | 1.25 | 1.25 | 1.25** | 1.25** | stop | stop |
| Quetiapine | 800 | 25, 100, 200, 300 | 800 | 600 | 500 | 400 | 300 | 200 | 150 | 100 | 75 | 50 | 25 | 12.5 | 12.5 | stop | stop | stop |
| Aripiprazole | 20 | 5,10, 15 | 20 | 17.5 | 15 | 12.5 | 10 | 10 | 7.5 | 7.5 | 5 | 5 | 2.5 | 2.5 | 2.5** | 2.5** | stop | stop |
| Haloperidol | 20 | 1, 5, 10 | 16 | 12 | 10 | 8 | 6 | 4 | 3 | 2 | 1 | 1 | 0.5 | 0.5 | 0.5** | 0.5** | stop | stop |
| Zuclopenthixol | 40 | 2, 10, 25 | 40 | 32 | 28 | 24 | 20 | 16 | 12 | 8 | 6 | 4 | 2 | 1 | 1 | 1** | 1** | stop |
| Sulpiride | 800 | 400, 50* | 800 | 600 | 500 | 450 | 400 | 350 | 300 | 250 | 200 | 150 | 100 | 50 | 50 | 50** | 50*** | stop |
| Paliperidone | 12 | 3*,6*,9* | 12 | 12** | 9 | 9** | 9** | 6 | 6 | 6** | 3 | 3 | 3** | 3** | 3*** | stop | stop | stop |
| Pimozide | 20 | 1, 4 | 20 | 16 | 12 | 10 | 8 | 6 | 4 | 3 | 2 | 1 | 1 | 0.5 | 0.5 | 0.5** | 0.5** | stop |
| Lurasidone | 148 | 18.5, 37, 74 | 148 | 111 | 92.5 | 92.5 | 74 | 74 | 55.5 | 55.5 | 37 | 37 | 18.5 | 18.5 | 9.25 | 9.25 | 9.25** | stop |
| Clozapine | 900 | 12.5*, 25, 100, 200 | 900 | 700 | 500 | 400 | 350 | 300 | 250 | 200 | 150 | 100 | 50 | 25 | 25 | 12.5 | 12.5 | stop |
| Amisulpride | 800 | 50, 100, 200, 400 | 800 | 700 | 600 | 500 | 400 | 350 | 300 | 250 | 200 | 150 | 100 | 50 | 25 | 25 | 25** | stop |
| Brexpiprazole | 4 | 4*,3*,2*,1* | 4 | 3 | 2 | 2 | 1 | 1 | 1** | 1** | 1*** | 1*** | 1*** | stop | stop | stop | stop | stop |
| Flupentixol | 18 | 0.5, 1, 3, 5 | 18 | 15 | 12 | 9 | 6 | 4 | 3 | 1.5 | 1 | 1 | 0.5 | 0.5 | 0.25 | 0.25 | 0.25** | stop |
| Cariprazine | 6 | 1.5*, 3*, 4.5*, 6* | 6 | 4.5 | 4.5 | 3 | 3 | 1.5 | 1.5 | 1.5** | 1.5** | 1.5*** | 1.5*** | 1.5*** | stop | stop | stop | stop |
| w = weeks  * undividable  ** 1 dose on alternate days  *** 1 dose every 4 days | | | | | | | | | | | | | | | | | | |

**Supplementary Material 2. Flowchart of participants in the PET study**

|  |
| --- |
| *Figure S1*. Flowchart of participants in the PET study. PA: former partial D_2_R agonist user, ANT: former D_2_R antagonist user. |

**Supplementary Material 3. Linear mixed effects model random and fixed effects results and marginalized means contrasts**

*Table S2.* Linear mixed effects model results the effect of discontinuation of partial D_2_R agonist versus D_2_R antagonist antipsychotics one week following discontinuation and at 6-8 weeks follow-up and controls on striatal D_2/3_R BP_ND_. There was no time by group interaction estimated for controls as these underwent only a single PET scan.

|  | Striatal D_2/3_R BP_ND_ | | |
| --- | --- | --- | --- |
| *Predictors* | *Estimates* | *CI* | *p-value* |
| Intercept | 3.98 | 3.73 – 4.22 | <0.001 |
| Group |  |  |  |
| *Antagonist users* | Reference group | | |
| *Partial agonist users* | -1.21 | -1.68 – -0.75 | <0.001 |
| *Controls* | -0.36 | -0.71 – -0.01 | 0.045 |
| Time | -0.10 | -0.51 – 0.30 | 0.608 |
| Time x Group  *Partial agonis users by 2-month follow-up   interaction* | 1.54 | 0.84 – 2.24 | <0.001 |
| Observations | 50 | | |
| Marginal R^2^ / Conditional R^2^ | 0.412 / 0.464 | | |

*Table S3.* Contrasts of the marginalized means of the LMEM of striatal D_2/3_R BP_ND_ in FEP patients who discontinued partial D_2_R agonist and D_2_R antagonist antipsychotics one week after discontinuation versus 6-8 weeks follow-up and controls.

| **contrast** | **estimate** | **SE** | **df** | **t-ratio** | **p-value** | **95% CI lower limit** | **95% CI upper limit** |
| --- | --- | --- | --- | --- | --- | --- | --- |
| Antagonist after 1 week vs. partial agonist after 1 week | 1.215 | 0.231 | 44.833 | 5.250 | <0.001 | 0.749 | 1.681 |
| Partial agonists after 1 week vs. controls | -0.856 | 0.233 | 44.833 | -3.668 | 0.001 | -1.326 | -0.386 |
| Antagonist after 1 week vs controls | 0.358 | 0.174 | 44.833 | 2.063 | 0.045 | 0.008 | 0.708 |
| Antagonist vs. partial agonist after 2 months | 0.324 | 0.282 | 44.993 | 1.147 | 0.257 | -0.245 | 0.893 |
| Partial agonist after 2 months vs. controls | 0.577 | 0.252 | 44.960 | 2.289 | 0.027 | 0.069 | 1.085 |
| Antagonist after 2 months vs. controls | 0.253 | 0.217 | 44.982 | 1.166 | 0.250 | -0.184 | 0.691 |
| Partial agonist after 1 week vs. after 2 months | -1.433 | 0.283 | 15.741 | -5.063 | <0.001 | -2.034 | -0.832 |
| Antagonist after 1 week vs. after 2 months | 0.105 | 0.209 | 21.784 | 0.502 | 0.621 | -0.329 | 0.539 |

*Table S4.* Linear mixed effects model results of annual relapse vs. no relapse in FEP patients one week following antipsychotic discontinuation and at 6-8 weeks follow-up and controls on striatal D_2/3_R BP_ND_.

|  | Striatal D_2/3_R BP_ND_ | | |
| --- | --- | --- | --- |
| *Predictors* | *Estimates* | *CI* | *p-value* |
| Intercept | 3.62 | 3.32 – 3.92 | <0.001 |
| Group |  |  |  |
| *Controls* | Reference group | | |
| *No relapse* | -0.27 | -0.78 – 0.25 | 0.301 |
| *Relapse* | 0.20 | -0.24 – 0.63 | 0.373 |
| Time | -0.15 | -0.76 – 0.46 | 0.629 |
| Time x Group |  |  |  |
| *No relapse by 2-month follow-up interaction* | 1.01 | 0.17 – 1.85 | 0.020 |
| Observations | 50 | | |
| Marginal R^2^ / Conditional R^2^ | 0.166 / 0.187 | | |

*Table S5.* Contrasts of the marginalized means of the LMEM of striatal D_2/3_R BP_ND_ in FEP patients with and without annual relapse who discontinued partial D_2_R agonist and D_2_R antagonist antipsychotics one week after discontinuation versus 6-8 weeks follow-up and controls.

| **contrast** | **estimate** | **SE** | **df** | **t-ratio** | **p-value** | **95% CI lower limit** | **95% CI upper limit** |
| --- | --- | --- | --- | --- | --- | --- | --- |
| No relapse after 1 week vs. relapse after 1 week | -0.462 | 0.258 | 44.985 | -1.789 | 0.080 | -0.983 | 0.058 |
| Controls vs. relapse after 1 week | -0.195 | 0.217 | 44.985 | -0.901 | 0.372 | -0.632 | 0.241 |
| Controls vs. no relapses after 1 week | 0.267 | 0.255 | 44.985 | 1.046 | 0.301 | -0.247 | 0.781 |
| Relapse after 2 months vs. no relapse after 2 months | -0.545 | 0.344 | 44.994 | -1.583 | 0.120 | -1.238 | 0.148 |
| Relapse after 2 months vs. controls | 0.048 | 0.314 | 44.986 | 0.154 | 0.878 | -0.584 | 0.681 |
| No relapse after 2 months vs. controls | 0.593 | 0.255 | 44.985 | 2.324 | 0.025 | 0.079 | 1.107 |
| Relapse after 1 week vs. after 2 months | 0.147 | 0.314 | 27.967 | 0.467 | 0.644 | -0.497 | 0.791 |
| No relapse after 1 week vs. after 2 months | -0.860 | 0.288 | 14.518 | -2.990 | 0.009 | -1.475 | -0.245 |

**Supplementary Material 4. Sensitivity analyses on exclusively the putamen**

**Supplementary Material 4A. Discontinuation of partial D_2_R agonist or D_2_R antipsychotics, and controls related to BP_ND_ of the putamen**

*Table S6.* Contrasts of the marginalized means of the LMEM of putamen D_2/3_R BP_ND_ in FEP patients who discontinued partial D_2_R agonist and D_2_R antagonist antipsychotics one week after discontinuation versus 6-8 weeks follow-up and controls,

| **contrast** | **estimate** | **SE** | **df** | **t-ratio** | **p-value** | **95% CI lower limit** | **95% CI upper limit** |
| --- | --- | --- | --- | --- | --- | --- | --- |
| Antagonist after 1 week vs. Partial agonist after 1 week | 1.087 | 0.306 | 46.481 | 3.554 | 0.001 | 0.471 | 1.702 |
| Partial agonists after 1 week vs. controls | -0.897 | 0.309 | 46.481 | -2.905 | 0.006 | -1.519 | -0.276 |
| Antagonist after 1 week vs controls | 0.189 | 0.242 | 46.481 | 0.781 | 0.439 | -0.298 | 0.677 |
| Antagonists vs. partial agonists after 2 months | -0.082 | 0.372 | 46.990 | -0.221 | 0.826 | -0.830 | 0.665 |
| Partial agonist after 2 months vs. controls | 0.351 | 0.328 | 46.807 | 1.069 | 0.291 | -0.309 | 1.011 |
| Antagonist after 2 months vs. controls | 0.269 | 0.302 | 46.965 | 0.890 | 0.378 | -0.339 | 0.876 |
| Partial agonist after 1 week vs. after 2 months | -1.248 | 0.351 | 16.035 | -3.558 | 0.003 | -1.991 | -0.504 |
| Antagonist after 1 week vs. after 2 months | -0.079 | 0.284 | 22.114 | -0.278 | 0.783 | -0.668 | 0.510 |

**Supplementary Material 4B. Relapse in FEP patients who discontinued partial D_2_R agonist or D_2_R antagonist antipsychotics, and controls related to BP_ND_ of the putamen**

*Table S7.* Contrasts of the marginalized means of the LMEM of putamen D_2/3_R BP_ND_ in FEP patients with and without annual relapse who discontinued partial D_2_R agonist and D_2_R antagonist antipsychotics one week after discontinuation versus 6-8 weeks follow-up and controls.

| **contrast** | **estimate** | **SE** | **df** | **t-ratio** | **p-value** | **95% CI lower limit** | **95% CI upper limit** |
| --- | --- | --- | --- | --- | --- | --- | --- |
| Relapse after 1 week vs. no relapse after 1 week | 0.213 | 0.323 | 45.496 | 0.658 | 0.514 | -0.437 | 0.862 |
| Relapse after 1 week vs controls | -0.067 | 0.269 | 45.496 | -0.250 | 0.804 | -0.609 | 0.475 |
| No relapses after 1 week vs. controls | -0.280 | 0.323 | 45.496 | -0.867 | 0.390 | -0.930 | 0.370 |
| No relapse after 2 months vs. relapse after 2 months | 0.717 | 0.403 | 46.876 | 1.779 | 0.082 | -0.094 | 1.528 |
| Relapse after 2 months vs. controls | -0.105 | 0.362 | 46.424 | -0.290 | 0.773 | -0.833 | 0.623 |
| No relapse after 2 months vs. controls | 0.612 | 0.323 | 45.496 | 1.897 | 0.064 | -0.038 | 1.262 |
| Relapse after 1 week vs. after 2 months | 0.038 | 0.335 | 22.230 | 0.112 | 0.912 | -0.656 | 0.731 |
| No relapse after 1 week vs. after 2 months | -0.892 | 0.317 | 13.797 | -2.811 | 0.014 | -1.574 | -0.211 |

**Supplementary Material 5. Sensitivity analyses on exclusively the caudate nucleus**

**Supplementary Material 5A. Discontinuation of partial D_2_R agonist or D_2_R antagonist antipsychotics, and controls related to BP_ND_ of the caudate nucleus**

*Table S8.* Contrasts of the marginalized means of the LMEM of the caudate nucleus D_2/3_R BP_ND_ in FEP patients who discontinued partial D_2_R agonist and D_2_R antagonist antipsychotics one week after discontinuation versus 6-8 weeks follow-up and controls,

| **contrast** | **estimate** | **SE** | **df** | **t-ratio** | **p-value** | **95% CI lower limit** | **95% CI upper limit** |
| --- | --- | --- | --- | --- | --- | --- | --- |
| Antagonist after 1 week vs. Partial agonist after 1 week | 1.148 | 0.247 | 46.840 | 4.643 | <.001 | 0.650 | 1.645 |
| Partial agonists after 1 week vs. controls | -0.777 | 0.250 | 46.840 | -3.111 | 0.003 | -1.279 | -0.274 |
| Antagonist after 1 week vs controls | 0.371 | 0.196 | 46.840 | 1.892 | 0.065 | -0.023 | 0.766 |
| Antagonists vs. partial agonists after 2 months | -0.166 | 0.301 | 46.996 | -0.552 | 0.584 | -0.773 | 0.440 |
| Partial agonist after 2 months vs. controls | 0.415 | 0.266 | 46.942 | 1.561 | 0.125 | -0.120 | 0.949 |
| Antagonist after 2 months vs. controls | 0.248 | 0.245 | 46.988 | 1.014 | 0.316 | -0.244 | 0.741 |
| Partial agonist after 1 week vs. after 2 months | -1.192 | 0.294 | 16.700 | -4.056 | 0.001 | -1.812 | -0.571 |
| Antagonist after 1 week vs. after 2 months | 0.123 | 0.236 | 23.435 | 0.520 | 0.608 | -0.365 | 0.610 |

**Supplementary Material 5B. Relapse in FEP patients who discontinued partial** **D_2_R agonist or D_2_R antagonist antipsychotics, and controls related to BP_ND_ of the caudate nucleus**

*Table S9.* Contrasts of the marginalized means of the LMEM of caudate nucleus D_2/3_R BP_ND_ in FEP patients with and without annual relapse who discontinued partial D_2_R agonist and D_2_R antagonist antipsychotics one week after discontinuation versus 6-8 weeks follow-up and controls.

| **contrast** | **estimate** | **SE** | **df** | **t-ratio** | **p-value** | **95% CI lower limit** | **95% CI upper limit** |
| --- | --- | --- | --- | --- | --- | --- | --- |
| Relapse after 1 week vs. no relapse after 1 week | 0.413 | 0.272 | 46.249 | 1.515 | 0.137 | -0.136 | 0.961 |
| Relapse after 1 week vs controls | 0.165 | 0.227 | 46.249 | 0.727 | 0.471 | -0.292 | 0.623 |
| No relapses after 1 week vs. controls | -0.248 | 0.272 | 46.249 | -0.908 | 0.368 | -0.796 | 0.301 |
| No relapse after 2 months vs. relapse after 2 months | 0.660 | 0.343 | 46.925 | 1.924 | 0.060 | -0.030 | 1.350 |
| Relapse after 2 months vs. controls | -0.056 | 0.308 | 46.683 | -0.182 | 0.856 | -0.677 | 0.564 |
| No relapse after 2 months vs. controls | 0.604 | 0.272 | 46.249 | 2.217 | 0.032 | 0.056 | 1.152 |
| Relapse after 1 week vs. after 2 months | 0.221 | 0.293 | 24.073 | 0.756 | 0.457 | -0.383 | 0.826 |
| No relapse after 1 week vs. after 2 months | -0.852 | 0.282 | 14.321 | -3.021 | 0.009 | -1.455 | -0.248 |
